# Climatic Drivers of Silicon Accumulation in a Model Grass Operate in Low- but Not High-Silicon Soils

**DOI:** 10.3390/plants12050995

**Published:** 2023-02-22

**Authors:** Scott N. Johnson, Rebecca K. Vandegeer, Justin O. Borevitz, Susan E. Hartley, David T. Tissue, Casey R. Hall

**Affiliations:** 1 Hawkesbury Institute for the Environment, Western Sydney University, Penrith, NSW 2751, Australia; 2 Research School of Biology, Australian National University, Canberra, ACT 2601, Australia; 3 School of Biosciences, University of Sheffield, Sheffield S10 2TN, UK

**Keywords:** false brome, phytoliths, precipitation, rainfall, seasonality, silica

## Abstract

Grasses are hyper-accumulators of silicon (Si), which is known to alleviate diverse environmental stresses, prompting speculation that Si accumulation evolved in response to unfavourable climatic conditions, including seasonally arid environments. We conducted a common garden experiment using 57 accessions of the model grass *Brachypodium distachyon*, sourced from different Mediterranean locations, to test relationships between Si accumulation and 19 bioclimatic variables. Plants were grown in soil with either low or high (Si supplemented) levels of bioavailable Si. Si accumulation was negatively correlated with temperature variables (annual mean diurnal temperature range, temperature seasonality, annual temperature range) and precipitation seasonality. Si accumulation was positively correlated with precipitation variables (annual precipitation, precipitation of the driest month and quarter, and precipitation of the warmest quarter). These relationships, however, were only observed in low-Si soils and not in Si-supplemented soils. Our hypothesis that accessions of *B. distachyon* from seasonally arid conditions have higher Si accumulation was not supported. On the contrary, higher temperatures and lower precipitation regimes were associated with lower Si accumulation. These relationships were decoupled in high-Si soils. These exploratory results suggest that geographical origin and prevailing climatic conditions may play a role in predicting patterns of Si accumulation in grasses.

## 1. Introduction

Most grasses take up and accumulate silicon (Si) more than any other inorganic constituent [1]. Si accumulation is increasingly recognised as playing an important functional role in plant ecology [2], particularly in terms of its role in relieving the adverse effects of environmental stress [3,4]. These include drought, heat, and salinity as well as biotic agents of plant stress (e.g., pathogens and herbivorous insects) [5]. The mechanisms for stress alleviation vary and are often incompletely understood, but usually involve silicification of tissues, which can have direct impacts (e.g., phytoliths that inhibit herbivory) [6] or indirect impacts on plant chemical and physiological processes (e.g., stomatal closure reducing transpiration losses) [7].

Given that Si has been reported to play an important role in the alleviation of drought stress in plants [8], a pervasive idea is that Si accumulation may be highly beneficial and therefore common in seasonally arid environments [9]. While Si accumulation may alleviate the impacts of drought, water limitation may in itself reduce the ability of plants to take up Si from the soil since passive uptake is highly dependent on the transpiration stream and stomatal conductance [10]. Water limitation has mostly been reported to limit Si accumulation in experimental situations, although some plant species appear to be able to maintain Si accumulation under drought/osmotic stress [11,12,13]. Maintaining, or even increasing, Si accumulation under these conditions may be achievable via increased evapotranspiration under hot and dry conditions [14]. Si accumulation is also attained via active, energy-dependent transport of Si [15,16,17,18], which may be less affected by water limitation.

Brightly, et al. [14] sampled 36 grass species grown under different temperature and irrigation regimes and generally found positive effects of high temperatures and water limitation on Si accumulation, although they concluded the relationships with water limitation were weak. In natural systems, Johnston, et al. [19] did not detect any patterns between precipitation and Si concentrations of grasses from Northern America. Using rain exclusion shelters, drought conditions increased Si concentrations in some grass species while decreasing them in others [20]. Sampling across a rainfall gradient, Katz, et al. [21] reported that Si (phytoliths) concentrations were positively correlated with water availability in a grass (*Avena sterilis*), but this was not seen in Asteraceae species. Sustained drought reduced Si accumulation in a barley (*Hordeum vulgare*) landrace and cultivar, whereas intermittent drought had little impact [22]. In short, consistent patterns between climatic variables and grass silicification have proved elusive. Moreover, climatic impacts on Si accumulation may be affected by the availability of bioavailable Si in the soil [23].

**Figure 1:**
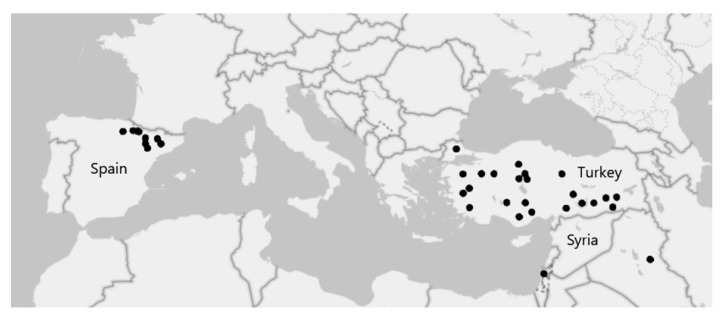
Map showing the location of the 57 *Brachypodium distachyon* accessions used in this research.

The objective of this study was to investigate potential relationships between geographic patterns of temperature and precipitation and Si accumulation in the model grass *Brachypodium distachyon*. Using a common garden experiment, we compared the Si accumulation capacity of 57 accessions with 19 climatic variables (Table S1) [24] for the Mediterranean region of origin (Figure 1). We hypothesised that accessions from seasonally arid regions have a greater capacity for Si uptake, which would be reflected in positive correlations with relevant temperature and precipitation climatic variables (e.g., precipitation seasonality). We predicted that these relationships may be altered by bioavailable Si in the soil.

## 2. Results

**Figure 2:**
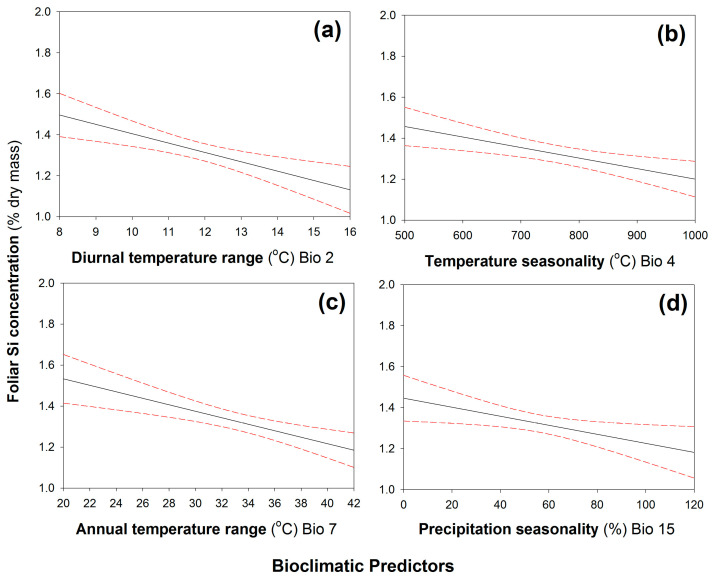
Significant negative correlations between leaf Si concentrations and (**a**) annual mean diurnal temperature range, (**b**) temperature seasonality, (**c**) annual temperature range, and (**d**) precipitation seasonality. N = 247. Regression line (solid line) fitted with 95% confidence displayed as dashed lines. See Table 1 for statistical analysis.

**Figure 3:**
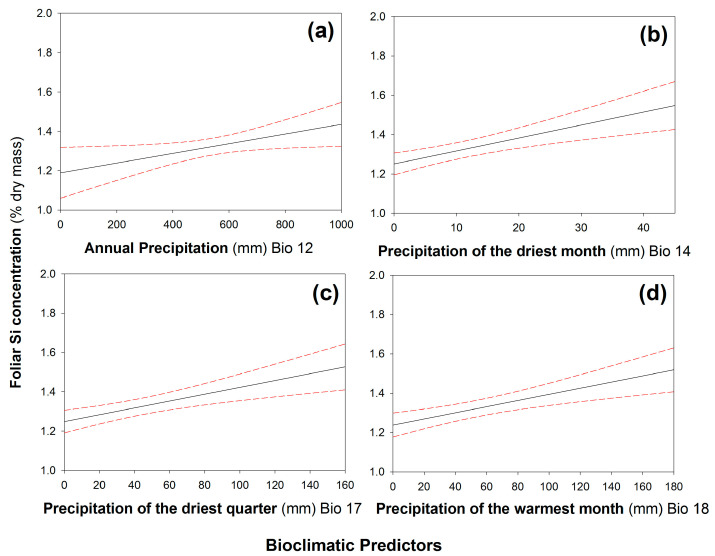
Significant positive correlations between leaf Si concentrations and (**a**) annual precipitation, (**b**) precipitation of the driest month, (**c**) precipitation of the driest quarter, and (**d**) precipitation of the warmest month. N = 247. Regression line (solid line) fitted with 95% confidence displayed as dashed lines. See Table 1 for statistical analysis.

When plants were grown under low-Si conditions, leaf Si concentrations were negatively correlated with four climatic variables (Table 1): annual mean diurnal temperature range (Figure 2a), temperature seasonality (Figure 2b), annual temperature range (Figure 2c), and precipitation seasonality (Figure 2d). In contrast, leaf Si concentrations were positively correlated with precipitation variables (Table 1): annual precipitation (Figure 3a), precipitation of the driest month (Figure 3b), precipitation of the driest quarter (Figure 3c), and precipitation of the warmest quarter (Figure 3d). When plants were grown under high-Si soil conditions, none of the climatic variables were correlated with leaf Si concentrations (Table S2).

**Figure 4:**
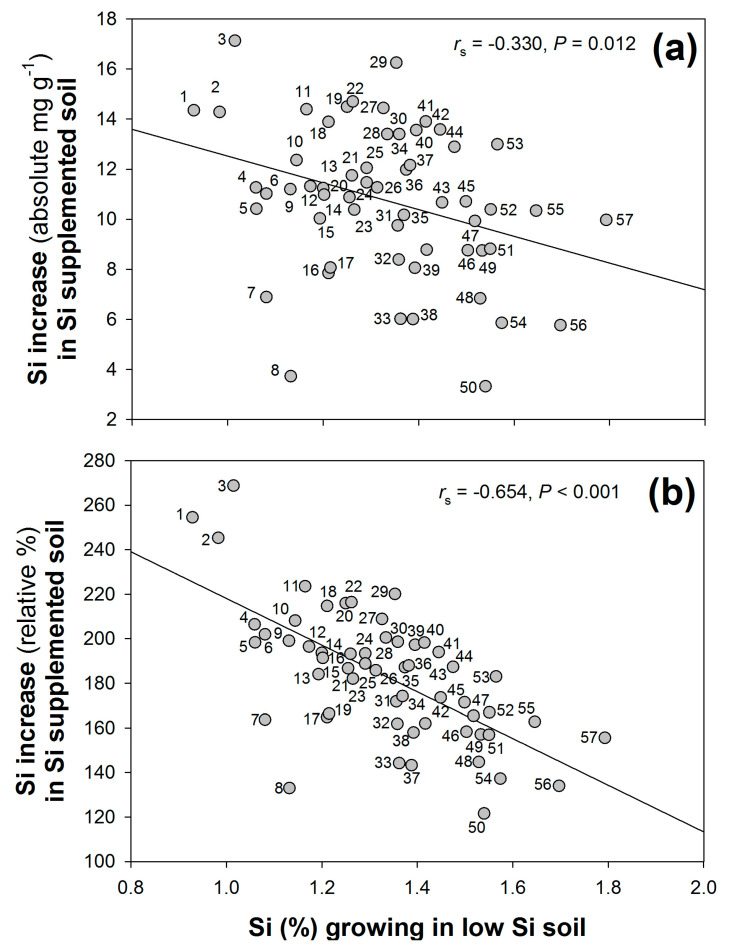
The relationship between shoot Si concentrations when growing in low-Si soil and the (**a**) absolute and (**b**) relative (%) increase in Si accumulation when accessions were grown in high (Si-supplemented) soil. Accessions indicated (1) Gaz2, (2) Adi6, (3) Adi15, (4) Bd23–01, (5) BdTR3a, (6) Mig3, (7) BdTR11a, (8) BdTR9a, (9) Bd3–1 RR 2009, (10) BdTR1k, (11) BdTR1d, (12) BdTR12a, (13) Adi1, (14) BdTR13c, (15) Bd3–1 Bd3–2, (16) BdTR11g, (17) Bd21–3 ANU, (18) Adi18, (19) Bd21, (20) Adi8, (21) BdTR10f, (22) BdTR1j, (23) Bd18–1, (24) Kah6, (25) Koz2, (26) Koz3, (27) Gaz6, (28) Kah4, (29) Kah5, (30) BdTR3s, (31) BdTR10i, (32) Bdis05–07, (33) BdTR2a, (34) BdTR10c, (35) Bd21–3 INRA, (36) Bdis28–08, (37) Adi16, (38) Gal1, (39) Cas2, (40) BdTR9f, (41) BdTR5m, (42) Bdis05–09, (43) BdTR3b, (44) BdTR2j, (45) Gaz3, (46) BdTR2p, (47) Bdis25–04, (48) Bdis22–06, (49) Bd25–01, (50) Bdis31–02, (51) Bd21 RR 2009, (52) Bdis31–01, (53) Bdis22–01, (54) Bdis32–02, (55) Bdis25–10, (56) BdTR1c, and (57) Pal2032.

Plants grown in high-Si soil had significantly higher concentrations of leaf Si compared with those grown in low-Si (2.41% ± 0.04 and 1.32% ± 0.02, respectively, mean ± standard error given) (H_1_ = 320.2, *p* < 0.001). There was, however, a significant negative correlation between this increase in Si concentrations, either in terms of absolute (Figure 4a) or percentage (Figure 4b) increase, and the Si concentrations observed when growing in low- (untreated) Si soil. In other words, accessions achieving relatively greater Si accumulation under low-Si conditions tended to show comparatively smaller increases in Si when the soil was supplemented with Si.

## 3. Discussion

Accessions of *B. distachyon* with the greatest capacity for Si accumulation under low-Si conditions tended to originate in regions with lower temperatures and higher precipitation patterns. It seems possible that water limitation hinders Si accumulation, which is potentially exacerbated by warmer temperatures. This refutes our hypothesis that accessions from seasonally arid regions would have the highest concentrations of Si. On the contrary, a climatic variable associated with seasonal aridity (precipitation seasonality) was negatively correlated with leaf Si concentrations. This relationship was decoupled, however, when we increased soil Si availability. 

### 3.1. Interpreting the Bioclimatic Variables

Many of the bioclimatic variables used in this study will correlate with one another given their similar nature; hence, we must be cautious about overinterpreting statistically significant correlations. What can we infer if we consider the strongest negative and positive correlations (*annual mean diurnal temperature range* and *precipitation of the warmest month*, respectively)? The first correlation reflects the range between minimum and maximum temperatures with larger values reflecting more extreme temperatures; the second correlation reflects the level of precipitation in the warmest three months (Table S1). Greater fluctuations in temperature might hinder Si accumulation because higher temperatures may exacerbate the effects of water limitation via evaporation from the soil surface [26], whereas colder temperatures may slow down Si absorption [27]. In contrast, higher amounts of precipitation during the active growing season (i.e., the warmest quarter) are likely to increase plant growth and nutrient uptake in general. This may explain why *B. distachyon* accessions from regions with low temperature fluctuations and ample precipitation during growth periods tended to accumulate more Si than accessions from regions with highly fluctuating temperatures and dry summers. Overall, increasing the availability of soil Si seems to mask this underlying capacity.

### 3.2. Linkages between Temperature, Precipitation, and Bioavailable Si in the Soil

Bioavailable Si derives from weathering and desilication of primary silicate minerals (lithogenic Si) or the deposition of silicified plant tissues (e.g., phytoliths) into the soil via plant litter (biogenic or phytogenic Si) [28]. In grassland and forest systems, the predominant source of silicic acid for plants is biogenic Si. In essence, Si is cycled from plant litter through the biogenic pool of soil Si and back to plants [29]. While higher temperatures generally increase Si release from phytoliths in the soil [30], this is inhibited in dry soils because soil water is required for dissolution [31]. Blecker, et al. [32] showed that soil biogenic Si (e.g., phytoliths) decreased with precipitation, proposing that faster soil phytolith turnover rates under higher rainfall regimes underpinned this relationship.

Quigley, et al. [23] also found that amorphous Si (pre-weathered silicates) in the soil was negatively correlated with precipitation. Moreover, they reported that dissolved Si (bioavailable silicic acid; H_4_SiO_4_) was also negatively correlated with precipitation, namely, concentrations were lowest under wetter conditions. If this was due to increased Si uptake by plants (i.e., depletion of dissolved soil Si), one would assume a negative relationship between dissolved soil Si and leaf Si concentrations, but, in fact, they reported a positive correlation between the two [23]. Considering multiple plant taxa, Cooke and Leishman [33] determined that soil Si availability does not generally correlate with Si accumulation, suggesting that other factors may influence these relationships. 

### 3.3. Accessions Differ in Their Capacity for Si Uptake

We observed that accessions achieving relatively higher Si accumulation under low-Si conditions attained smaller increases in Si, when supplemented with Si, compared with accessions with lower Si concentrations that showed stronger patterns of Si uptake when it was made more available. A possible interpretation of this result is that some *B. distachyon* accessions are well adapted to Si accumulation under low bioavailable Si conditions, whereas other accessions show greater plasticity to Si availability in the soil. When compost was supplemented with Si (without drought exposure), Thorne, et al. [13] observed that ‘high-Si accumulator’ wheat (*Triticum aestivum*) landraces achieved similar increases in Si accumulation to ‘low-Si accumulator’ landraces. In contrast, when grown hydroponically (without osmotic stress) the ‘high-Si accumulators’ showed disproportionately larger increases in shoot Si concentrations compared with ‘low-Si accumulators’ [13]. This suggests that the soil itself places some constraints on the plasticity of Si accumulation, which is also affected by factors such as root exudation [34].

### 3.4. Do Trade-Offs Operate in Water Limited Environments?

Many studies show that nutrient limitation promotes Si accumulation [35,36]. Quigley, et al. [35] also observed a leaf-level trade-off between Si and carbon, which was stronger at arid sites than mesic sites. They concluded that plants used Si rather than invest in C-based leaf construction and C fixation, which is relatively costly when water is limited. Si accumulation may not necessarily be higher in plants growing in arid conditions because of physiological constraints; therefore, it remains possible that Si could play a relatively more important role in arid regions. Si-reinforced cell walls may prevent cell collapse during water shortages, which could be beneficial in seasonally arid environments [9]. 

## 4. Materials and Methods

### 4.1. Common Garden Experiment

Fifty-seven *Brachypodium distachyon* accessions (Table S3) were used. These were mainly obtained from the Australian National University (Canberra, ACT, Australia) with additional accessions from Western Sydney University (Richmond, NSW, Australia). Germplasm was originally collected from its native Mediterranean region [37] (Figure 1). The accessions were grown from seed in soil recovered from the Hawkesbury Campus of Western Sydney University (33.6138° S, 150.7500° E). Seeds were initially stratified for five days at 4 °C before planting. Plants were grown in a 50:50 composite of sandy loam and loam soil with low bioavailable Si levels of 16.00 mg kg^−1^ (extracted with CaCl_2_ [38]); see Table S4 for further soil chemistry. The experiment involved growing half of the plants in ‘low-Si soil’ (−Si) and half in ‘high-Si soil’ (+Si). To obtain high-Si soil, half of the pots were watered with a solution consisting of potassium silicate (Agsil32, PQ Australia, SA, Australia) at a concentration of 2 mM (SiO_2_ equivalent) and adjusted to pH 7 using HCl. The low-Si soil pots were watered with a control solution containing 1.6 mM KCl to balance additional K^+^ and Cl^−^ in the Si-supplemented soils; see [39] for details.

Plants were grown in a growth cabinet (Climatron, Thermoline Scientific) maintained at 22/16 °C on an 8:16 h light:dark cycle. The humidity was kept at ca. 60% and light levels were ca. 150 μmol m^−2^ s^−1^. Plants were watered with 25 mL of either −Si or +Si solutions (low- and high-Si soil, respectively) every 10 days for the first 10 weeks of the experiment and then every 7 days for the remaining four weeks of the experiment for a total of 14 weeks. Plants were harvested, oven dried, and analysed for leaf Si concentrations at this point. There was a median of five replicates for each accession under low- and high-Si soil conditions at the end of the experiment. We obtained 19 climatic variables (temperature and precipitation; Table S1) for each accession from the WorldClim database [24] to examine any relationships between leaf Si accumulation and climatic variables (see Table S1). 

### 4.2. Leaf Si Accumulation

We determined leaf Si concentrations using ca. 100 mg of ground shoot tissue placed into small receptacles (Malvern PANalytical, Malvern UK), which were then analysed with an X-ray fluorescence spectrometer (Epsilon 3×, PANalytical) using the procedure and certified reference material described in Hiltpold et al. [40]. This method was based on the methodology developed by Reidinger, et al. [41].

### 4.3. Statistical Analysis

Relationships between leaf concentrations of Si and the 19 climatic variables (Table S1) were examined for all plants grown under low- and high-Si soil conditions separately using Spearman’s correlation tests. To account for repeated tests, which can increase false-positive (significance) rates, we adjusted *p* values using the Benjamini and Hochberg False Discovery Method [25]. Differences in leaf Si concentrations between plants grown under low- and high-Si soil conditions were examined with a Kruskal–Wallis test. Relationships between Si concentrations in plants grown in low-Si soil and corresponding increases in Si concentrations when growing in high-Si soil were conducted using mean values for each accession. All analyses were conducted in Genstat version 21 (VSN International Ltd., Hemel Hempstead, UK).

## 5. Conclusions

This exploratory study suggests that *B. distachyon* accessions from regions with higher precipitation during the warmest months have a greater capacity for Si accumulation than those from drier regions. Warmer temperatures and adequate rainfall are prerequisites for the dissolution of phytoliths [28,29], which may go some way to explaining this result. Given the important functional role of Si accumulation in many grasses, understanding how climatic factors affect patterns of Si uptake and accumulation may become increasingly important as the planet experiences environmental change at an unprecedented rate. 

## Figures and Tables

**Table 1:** Correlation test results for leaf Si concentrations and climatic variables for plants grown under low-Si soil conditions. *p* values corrected to account for multiple testing using the Benjamini and Hochberg False Discovery method [25].

Statistical Significance	Correlation	Climatic Variable	Figure	*r_s_*	*p*
Significant	Negative	Bio 2—Annual Mean Diurnal Temperature Range	2a	−0.262	< 0.001
		Bio 4—Temperature Seasonality (Standard Deviation)	2b	−0.188	0.010
		Bio 7—Annual Temperature Range	2c	−0.210	0.010
		Bio 15—Precipitation Seasonality	2d	−0.156	0.033
	Positive	Bio 12—Annual Precipitation	3a	0.186	0.019
		Bio 14—Precipitation of Driest Month	3b	0.187	0.014
		Bio 17—Precipitation of Driest Quarter	3c	0.184	0.011
		Bio 18—Precipitation of Warmest Quarter	3d	0.192	0.011
Non-significant	No correlation	Bio 1—Annual Mean Temperature		0.073	0.319
		Bio 3—Isothermality		0.068	0.338
		Bio 5—Max Temperature of Warmest Month		−0.116	0.119
		Bio 6—Min Temperature of Coldest Month		0.139	0.061
		Bio 8—Mean Temperature of Wettest Quarter		0.048	0.506
		Bio 9—Mean Temperature of Driest Quarter		−0.078	0.303
		Bio 10—Mean Temperature of Warmest Quarter		−0.001	0.999
		Bio 11—Mean Temperature of Coldest Quarter		0.125	0.095
		Bio 13—Precipitation of Wettest Month		0.102	0.174
		Bio 16—Precipitation of Wettest Quarter		0.080	0.310
		Bio 19—Precipitation of Coldest Quarter		0.039	0.575

## Data Availability

The dataset associated with this research will be made available via the FigShare repository [details to follow pending review].

## Supplementary Materials

The following supporting information can be downloaded at: https://www.mdpi.com/article/10.3390/plants12050995/s1, Table S1: The 19 climatic variables used in the study. Definitions and interpretations summarises descriptions outlined in O’Donnell and Ignizio (2012); Table S2: Correlation test results for foliar Si concentrations and climatic variables for plants grown in high Si soils (+Si); Table S3: Collection locations for the *Brachypodium distachyon* accessions; Table S4: Characteristics of the homogenised soil used in the common garden experiment. References [42,43,44] are cited in the supplementary file.
