# Supplementary material for: Climatic Drivers of Silicon Accumulation in a Model Grass Operate in Low- but Not High-Silicon Soils
Source: Plants (Basel). 2023 Feb 22;12(5):995. doi: 10.3390/plants12050995 (PMC10005694; doi:10.3390/plants12050995)
Supplement: Supplementary file 1 [file plants-12-00995-s001.zip › plants-2196201-supplementary.pdf]

Supplemental Material

Climatic drivers of silicon accumulation in a model grass operate in low but not high silicon soils

Scott N. Johnson, Rebecca K. Vandegeer, Justin O. Borevitz, Susan E. Hartley, David, T. Tissue & Casey R. Hall

Table S1. The 19 climatic variables used in the study. Definitions and interpretations summarises descriptions outlined in O'Donnell and Ignizio (2012).

Bio	Variable	Definition	Units	Interpretation
1	Annual mean temperature	The annual mean temperature.	ºC	The annual mean temperature approximates the total energy inputs for an ecosystem.
2	Annual mean temperature diurnal range	The diurnal range is the difference between that month's maximum and minimum temperature.	ºC	This index can help provide information pertaining to the relevance of temperature fluctuation. Larger values potentially exacerbate the effects of water limitation at the higher temperature and nutrient (Si) uptake at the lower temperature.
3	Isothermality	Isothermality quantifies how large the day-to-night temperatures oscillate relative to the summer-to-winter (annual) oscillations.	%	Isothermality is used to quantify the difference between how much temperature varies on a daily basis versus how much it varies over a year. Low isothermality occurs when day-to-night temperature swings are similar in magnitude to the annual temperature changes.
4	Temperature seasonality (standard deviation)	The amount of temperature variation over a given year (or averaged years) based on the standard deviation (variation) of monthly temperature averages.	ºC	Temperature seasonality is a measure of temperature change over the course of the year. The larger the standard deviation, the greater the variability of temperature, which may exacerbate water limitation and nutrient uptake.
5	Maximum temperature of warmest month	The maximum monthly temperature occurrence over a given year (time-series) or averaged span of years (normal).	ºC	This variable identifies warm temperature anomalies throughout the year.

Table S1. *Continued*

Bio	Variable	Definition	Units	Interpretation
6	Minimum temperature of coldest month	The minimum monthly temperature occurrence over a given year (time-series) or averaged span of years (normal).	ºC	This variable identifies cold temperature anomalies throughout the year.
7	Annual temperature range	A measure of temperature variation over a given period.	ºC	This variable quantifies ranges of extreme temperature conditions. Larger temperature ranges potentially exacerbate water limitation and nutrient uptake.
8	Mean temperature of wettest quarter	This quarterly index approximates mean temperatures that prevail during the wettest season.	ºC	This variable provides mean temperatures during the wettest three months of the year.
9	Mean temperature of driest quarter	This quarterly index approximates mean temperatures that prevail during the driest quarter.	ºC	This variable provides mean temperatures during the driest three months of the year.
10	Mean temperature of warmest quarter	This quarterly index approximates mean temperatures that prevail during the warmest quarter	ºC	This variable provides mean temperatures during the warmest three months of the year.

Table S1. *Continued*

Bio	Variable	Definition	Units	Interpretation
11	Mean temperature of the coldest month	This variable approximates mean temperatures that prevail during the coldest quarter.	ºC	This variable provides mean temperatures during the coldest three months of the year.
12	Annual precipitation	This is the sum of all total monthly precipitation values.	mm	Annual total precipitation approximates the total water inputs. Higher values potentially facilitate plant growth and nutrient uptake.
13	Precipitation of wettest month	This variable identifies the total precipitation that prevails during the wettest month	mm	Useful for in the context of extreme precipitation conditions during the year.
14	Precipitation of the driest month	This variable identifies the total precipitation that prevails during the driest month.	mm	Useful in the context of extreme precipitation conditions during the year. Lower values potentially limit nutrient uptake and transpiration during the driest part of the year.
15	Precipitation seasonality (CV)	This is a measure of the variation in monthly precipitation totals over the course of the year.	%	This reflects variability in precipitation, this index provides a percentage of precipitation variability where larger percentages represent greater variability of precipitation. Greater variability potentially increases drought events which may limit nutrient uptake.

Table S1. *Continued*

Bio	Variable	Definition	Units	Interpretation
16	Precipitation of the wettest quarter	This variable approximates total precipitation that prevails during the wettest quarter.	mm	This variable provides total precipitation during the wettest three months of the year.
17	Precipitation of driest quarter	This quarterly variable approximates total precipitation that prevails during the driest quarter.	mm	The variable provides total precipitation during the driest three months of the year. Lower values potentially limit nutrient uptake and transpiration during this dry period.
18	Precipitation of the warmest quarter	This quarterly variable approximates total precipitation that prevails during the warmest quarter.	mm	This variable provides total precipitation during the warmest three months of the year. Lower values potentially limit nutrient uptake and transpiration during warm periods.
19	Precipitation of the coldest quarter	This quarterly variable approximates total precipitation that prevails during the coldest quarter.	mm	This variable provides total precipitation during the coldest three months of the year.

Table S2. Correlation test results for foliar Si concentrations and climatic variables for plants grown in high Si soils (+Si). *P* values corrected to account for multiple testing using the Benjamini and Hochberg False Discovery Method (Benjamini and Hochberg 1995).

**Climatic variable**	r_s	***P***
Bio 1—Annual Mean Temperature	0.093	0.874
Bio 2—Annual Mean Diurnal Temperature Range	–0.058	0.843
Bio 3—Isothermality	0.012	0.999
Bio 4—Temperature Seasonality (Standard Deviation)	–0.050	0.806
Bio 5—Max Temperature of Warmest Month	0.032	0.999
Bio 6—Min Temperature of Coldest Month	0.147	0.171
Bio 7—Annual Temperature Range	–0.064	0.972
Bio 8—Mean Temperature of Wettest Quarter	–0.004	0.999
Bio 9—Mean Temperature of Driest Quarter	0.001	0.988
Bio 10—Mean Temperature of Warmest Quarter	0.060	0.922
Bio 11—Mean Temperature of Coldest Quarter	0.148	0.342
Bio 12—Annual Precipitation	0.030	0.992
Bio 13—Precipitation of Wettest Month	0.083	0.865
Bio 14—Precipitation of Driest Month	0.009	0.999
Bio 15—Precipitation Seasonality	0.030	0.930
Bio 16—Precipitation of Wettest Quarter	0.069	0.999
Bio 17—Precipitation of Driest Quarter	0.012	0.999
Bio 18—Precipitation of Warmest Quarter	–0.004	0.999
Bio 19—Precipitation of Coldest Quarter	0.052	0.855

Table S3. Collection locations for the *Brachypodium distachyon* accessions.

Accession name	Latitude	Longitude	Elevation (m)
Adi1	38.35228	37.77069	1744.1
Adi15	38.35228	37.77069	1744.1
Adi16	38.35228	37.77069	1744.1
Adi18	38.35228	37.77069	1744.1
Adi6	38.35228	37.77069	1744.1
Adi8	38.35228	37.77069	1744.1
Bd18-1	39.36785	33.71974	1101.1
Bd21	33.76088	44.40308	42.1
Bd21 RR 2009	33.76088	44.40308	42.1
Bd21-3 ANU	33.76088	44.40308	42.1
Bd21-3 INRA	33.76088	44.40308	42.1
Bd23-01	33.76088	44.40308	42.1
Bd25-01	32.72841	35.04613	518.4
Bd3-1 Bd3-2	33.76088	44.40308	42.1
Bd3-1 RR 2009	33.76088	44.40308	42.1
Bdis05-07	41.78622	-0.18278	293.2
Bdis05-09	41.78622	-0.18278	293.2
Bdis22-01	42.61005	-0.89319	596.7
Bdis22-06	42.61005	-0.89319	596.7
Bdis25-04	42.63651	-1.30484	435.6
Bdis25-10	42.63651	-1.30484	435.6
Bdis28-08	42.58087	-2.20273	465.5
Bdis31-01	42.09763	0.87750	496.6
Bdis31-02	42.09763	0.87750	496.6
Bdis32-02	42.09763	0.87750	496.6
BdTR10c	37.77823	31.88491	1286.5
BdTR10f	37.76155	39.58242	1158.2
BdTR10i	37.45880	41.24418	979.6
BdTR11a	38.41678	28.03132	980.7
BdTR11g	41.42163	27.47689	124.8
BdTR12a	36.78303	32.96298	1960.3
BdTR13c	39.41286	32.98812	1193.7
BdTR1c	39.73816	28.04020	365.3
BdTR1d	38.76411	28.58512	607.2
BdTR1j	37.42729	28.58521	515.2
BdTR1k	39.75949	29.67804	1012.3

Table S3. *Continued*

Accession name	Latitude	Longitude	Elevation (m)
BdTR2a	39.75295	30.78863	932.3
BdTR2j	39.41286	32.98812	1193.7
BdTR2p	39.75445	33.53788	869.8
BdTR3a	37.76786	33.51961	1013.1
BdTR3b	37.10885	34.07141	2387.2
BdTR3s	38.09542	40.65857	687.1
BdTR5m	40.39365	32.98537	1601.6
BdTR9a	39.74759	36.81579	1419.3
BdTR9f	37.42729	28.58521	515.2
Cas2	41.46507	0.01765	233.5
Gal1	42.56319	-0.78688	631.4
Gaz2	37.39081	37.12772	584.9
Gaz3	37.39081	37.12772	584.9
Gaz6	37.39081	37.12772	584.9
Kah4	37.73397	38.53339	666.0
Kah5	37.73397	38.53339	666.0
Kah6	37.73397	38.53339	666.0
Koz2	38.15229	41.60967	864.2
Koz3	38.15229	41.60967	864.2
Mig3	42.14799	-0.19497	570.2
Pal2032	41.76075	1.21082	400.0

Table S4. Characteristics of the homogenised soil used in the common garden experiment. Mean ± standard error shown (N = 3). Analysis conducted on oven dried (40ºC) soil sieved < 2mm by Environmental Analysis Laboratory, Southern Cross University, Australia. Specific methods described in Rayment and Lyons (2011); bioavailable silicon was extracted with $CaCl_2$ (Sauer et al. 2006); phosphorous and potassium levels were determined using a variation of the method described by Morgan (1941). Inhouse method: S10 https://www.scu.edu.au/environmental-analysis-laboratory---eal/

**Soil characteristic**	**Common garden**
pH	5.80 ± 0.22
Bioavailable silicon (mg kg^{-1})	16.00 ± 3.46
Phosphorus (mg kg^{-1})	16.84 ± 1.51
Nitrate nitrogen (mg kg^{-1})	11.46 ± 6.54
Ammonium nitrogen (mg kg^{-1})	2.29 ± 0.52
Potassium (mg kg^{-1})	115.34 ± 24.92
Total carbon (%)	1.04 ± 0.29
Total nitrogen (%)	0.08 ± 0.02
Estimated organic matter (%)	1.83 ± 0.50

Literature Cited in Supplemental Material

Benjamini, Y., and Y. Hochberg. 1995. Controlling the false discovery rate: A practical and powerful approach to multiple testing. Journal of the Royal Statistical Society. Series B (Methodological) **57**:289-300.

Morgan, M. F. 1941. Chemical soil diagnosis by the universal soil testing system. Connecticut Agricultural Experiment Station Circular **450**:579-628.

O'Donnell, M. S., and D. A. Ignizio. 2012. Bioclimatic predictors for supporting ecological applications in the conterminous United States. U.S. Geological Survey Data Series 691, Reston, Virginia, USA.

Rayment, G. E., and D. J. Lyons. 2011. Soil Chemical Methods - Australasia. CSIRO Publishing, Collingwood, VIC.

Sauer, D., L. Saccone, D. J. Conley, L. Herrmann, and M. Sommer. 2006. Review of methodologies for extracting plant-available and amorphous Si from soils and aquatic sediments. Biogeochemistry **80**:89-108.
